# The Role of FoxC2 Transcription Factor in Tumor Angiogenesis

**DOI:** 10.1155/2012/204593

**Published:** 2011-11-16

**Authors:** Tsutomu Kume

**Affiliations:** Feinberg Cardiovascular Research Institute, Feinberg School of Medicine, Northwestern University, 303E Chicago Avenue, Chicago, IL 60611, USA

## Abstract

Much has been learned about the mechanisms underlying tumor angiogenesis, and therapies that target vascular endothelial growth factor (VEGF) to limit tumor angiogenesis and subsequent disease progression have recently been approved. However, the transcriptional mechanisms that regulate pathological angiogenesis remain largely unknown. FoxC2, a member of the Forkhead box (Fox) transcription factor family, is critical for vascular formation during development, and recent studies have shown that FoxC2 is expressed in the endothelium of tumors in both humans and mice. In a B16 mouse melanoma model, Foxc2 deficiency reduced tumor growth and neovascularization and was associated with impairments in mural-cell coverage and increases in endothelial-cell apoptosis in tumor blood vessels. FoxC2 is also expressed by tumor cells in human breast, colonic, and esophageal cancer and participates in the epithelial-mesenchymal transition (EMT), a key process that leads to the invasion and metastasis of aggressive tumors. Collectively, these observations suggest that FoxC2 is essential for tumor angiogenesis and disease progression and that FoxC2 may be a viable target for cancer therapy.

## 1. Introduction

Tumor angiogenesis is a crucial contributor to tumor development and progression [1] because tumor blood vessels supply oxygen and nutrients to the tumor tissue and facilitate cancer cell invasion and metastasis. Tumor vessels are usually disorganized and excessively branched [2–4], and formation of the tumor vasculature involves complex interactions between tumor cells and their microenvironment that are controlled by numerous angiogenic factors secreted from both tumor cells and other cells in the tumor microenvironment. The mechanisms by which proangiogenic factors such as vascular endothelial growth factor (VEGF) promote tumor angiogenesis have been intensively studied [2–4]; however, the transcription factors that participate in this process remain incompletely understood. The FoxC2 transcription factor is a newly recognized regulator of tumor angiogenesis and metastasis, and this paper summarizes what is currently known about the role of FoxC2 in cancer.

## 2. Forkhead Transcription Factors in Cancer

The Fox (Forkhead box) transcription factors form a large family of proteins with similar DNA-binding domains that are evolutionarily conserved from yeast to humans [5–7]; other functional regions of Fox proteins, such as the transactivation and transrepression domains, are largely divergent. The consensus, seven-nucleotide core sequence bound by monomeric Fox proteins is 5′-(G/A)(T/C)(A/C)AA(C/T)A-3′ [8, 9], and Fox proteins are distributed among 19 subfamilies (FoxA to FoxS) based on similarities in their DNA-binding domains. Uppercase letters are used to designate human Fox genes (e.g., FoxC2), the first letter is capitalized for mouse genes (e.g., FoxC2), and the first and subclass letters are capitalized for chordates (e.g., FoxC2).

Fox proteins have many essential roles in embryonic development, and the mutation or dysregulation of FOX genes is often associated with disease, including cancer [10]. FoxM1 is expressed in glioma and pancreatic cancer cells and promotes angiogenesis by regulating VEGF expression [11, 12], FoxO1/3/4 act as tumor suppressors in prostate cancer and leukemia [13, 14], and recent evidence suggests that FoxC2 is a key factor in tumor development and disease progression. Thus, the elucidation of *FOX* gene function will likely identify new strategies for the treatment of cancer.

## 3. Role of FoxC2 in Vascular Development and Angiogenesis

Murine FoxC2 is highly expressed in blood endothelial cells during embryonic development [16–19]. FoxC2 contributes to arterial cell specification in the early developing embryo as a downstream regulator of the Notch signaling pathway [18, 20–22], and FoxC2 participates in angiogenesis [23, 24] through the activity of its downstream targets CXC-chemokine receptor 4 (CXCR4) and integrin *β*3 [25, 26]. The transcriptional activity of FoxC2 in vascular endothelial cells is modulated by VEGF signaling [20], and FoxC2 is critical for the migration of endothelial cells toward VEGF or stromal-cell-derived factor 1 (SDF-1) and for the formation of microvessels in vitro [25, 26]. Recent studies have shown that the activity of FoxC2 and the Ets transcription factor Etv2 combine to regulate endothelial-specific gene expression during early development, including Flk1, Tie2, and VE-cadherin [27], and the Ets transcription factors are known to be important for tumor angiogenesis [28], but whether FoxC2 and Etv2 also coregulate tumor vessel formation has yet to be determined.

The importance of epigenetic changes during the regulation of angiogenesis, including the methylation or histone acetylation of angiogenesis-associated genes, is becoming increasingly apparent [29]; for example, the histone deacetylase (HDAC) SIRT1 induces the sprouting and branching of endothelial cells by deacetylating Foxo1 [30]. SIRT1 also regulates the angiogenic activity of endothelial cells by deacetylating the Notch1 intracellular domain (NICD) [31], and a Notch transcriptional activation complex containing the NICD physically and functionally interacts with FoxC2 [20]. Collectively, these observations suggest that FoxC2 may be involved in SIRT1-mediated transcriptional control of angiogenesis.

FoxC2 is also expressed in lymphatic endothelial cells during development [32, 33], and mutations in human FoxC2 are responsible for the autosomal dominant syndrome lymphedema distichiasis, which is characterized by the obstruction of lymph drainage in the limbs and by the growth of an extra set of eyelashes [34, 35]. Congenital lymphatic defects are also observed in FoxC2 mutant mice: heterozygous FoxC2 mutants display hyperplasia of the lymphatic vessels [36], and homozygous FoxC2 mutants have defective lymphatic valves and abnormal pericyte recruitment of lymphatic vessels [33]. The contribution of FoxC2 to lymphangiogenesis in pathological conditions such as cancer remains unknown.

## 4. FoxC2 in Tumor Angiogenesis

**Figure 1:**
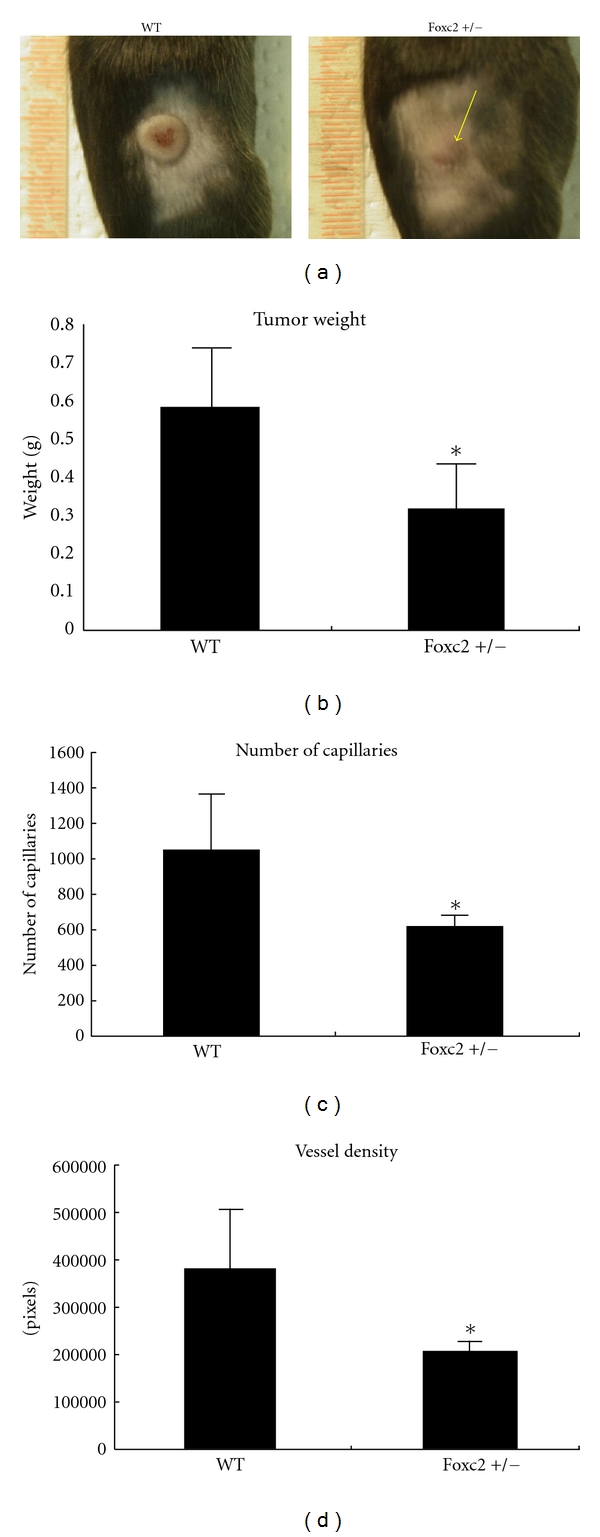
Tumor development and angiogenesis are impaired in FoxC2+/– mice. (a) Subcutaneous growth of B16 melanoma cells in WT and FoxC2+/– mice 11 days after injection. The yellow arrow identifies a smaller tumor in a FoxC2+/– mouse. (b) A difference in tumor weight between WT and FoxC2+/– mice was observed 11 days after the injection of B16 melanoma cells. The data are from five independent experiments and are presented as mean ± SD. Statistical significance was evaluated with Student's *t*-test. *P < 0.05 versus WT. (c, d) Tumor angiogenesis is reduced in FoxC2+/– mice. (c) The total number of capillaries was calculated by counting PECAM-1-positive endothelial cells in B16 tumors. Statistical significance was evaluated with Student's *t*-test (*P < 0.05 versus WT). (d) Total vessel density was calculated by measuring the evaluated with the Student's *t*-test PECAM-1-positive vessel area. Statistical significance was evaluated with Student's *t*-test. *P < 0.05 versus WT. Adapted from Sano et al. [15].

FoxC2 expression has been detected in the tumor endothelium of both human and mouse melanomas, which suggests that FoxC2 has a role in tumor angiogenesis (Table 1) [15]. Homozygous mutations of FoxC2 lead to perinatal lethality, so the role of FoxC2 in melanoma was confirmed by subcutaneously implanting mouse B16 melanoma cells in wild-type and heterozygous FoxC2 mutant (FoxC2+/–) mice. Tumor growth and angiogenesis were remarkably lower in the FoxC2+/– mutants (Figure 1) [15], which confirms that FoxC2 has an essential role in tumor angiogenesis. Matrix metalloproteinase (MMP) 2 expression was also diminished in B16 tumors from FoxC2+/– mice [15], which is consistent with evidence that MMP2 is important for tumor growth and angiogenesis [37, 38]. The expression of the lymphatic endothelial markers Prox1 and Lyve1 were unchanged [15], despite evidence that FoxC2 is expressed in lymphatic endothelial cells. 

VEGF-A expression was also impaired in B16 tumors from FoxC2+/– mice [15], and this decline likely limits tumor neovascularization. The cells responsible for the decline in VEGF-A expression have yet to be identified; however, autocrine VEGF signaling promotes endothelial cell survival [42], and smooth muscle *α*-actin- (*α*SMA-) positive cancer-associated fibroblasts (CAFs), which are known to express VEGF-A in the tumor microenvironment [43–45], were less common in tumors from FoxC2+/– mice than in tumors from wild-type mice [15]. CAFs also secrete stromal cell-derived factor 1 (SDF-1), which stimulates tumor growth by activating its receptor, CXCR4, on tumor cells and by functioning as a chemoattractant for the recruitment of bone-marrow-derived endothelial progenitor cells that subsequently promote angiogenesis [45]. FoxC2 is also known to regulate the CXCR4-dependent mobilization of endothelial cells and bone-marrow-derived endothelial progenitor cells [25, 46], so FoxC2 might have a bimodal influence on the contribution of bone-marrow-derived cells to tumor angiogenesis by increasing both cell mobilization and the SDF-1-mediated recruitment of mobilized cells to tumors. Furthermore, FoxC2 expression is induced by hypoxia after ischemia/reperfusion injury in the kidney [47], and the hypoxic gradient that develops in the tumor microenvironment increases SDF-1 expression in CAFs and CXCR4 expression in tumor cells [48, 49]; thus, FoxC2 could also contribute to tumor angiogenesis through a hypoxia-related mechanism. 

Fibroblasts in the tumor microenvironment are a heterogeneous cell population, and the conventional markers *α*SMA and vimentin cannot identify all CAFs [50]. Furthermore, transforming growth factor- (TGF-)  *β*1 can induce the phenotypic conversion of proliferating vascular endothelial cells into fibroblast-like cells; this endothelial-to-mesenchymal transition (EndMT) takes place at the invasive front of tumors in mice [51], and, consequently, antiangiogenic treatments may limit cancer progression, in part, by directly influencing the EndMT in tumor vessels. The molecular mechanisms and signaling pathways that control EndMT have yet to be elucidated; however, CAFs can develop directly from cancer cells through the epithelial-to-mesenchymal transition (EMT) [52], which is regulated by FoxC2 (as discussed in the next section). CAFs can also evolve from resident fibroblasts, smooth muscle cells, pericytes, or bone-marrow-derived mesenchymal stem cells through a mesenchymal-to-mesenchymal transition (MMT), which is regulated by tumor-cell-derived factors such as TGF-*β*, platelet-derived growth factor (PDGF), and basic fibroblast growth factor (FGF) [53]; whether FoxC2 participates in the MMT is unknown.

The declines in tumor growth and vascularity observed in FoxC2+/– mice were also associated with abnormally low levels of PDGF-B, which is likely attributable to the impaired growth of smooth muscle cells surrounding the tumor blood vessels [15]. The precise role of PDGF signaling in tumor angiogenesis remains a subject of debate [54]; however, PDGF mediates the maturation and stabilization of blood vessels during normal vessel growth by regulating the interaction between endothelial cells and mural cells (e.g., vascular smooth muscle cells and pericytes) that are loosely associated with the tumor endothelium, and PDGF-B inhibition is associated with both the loss of mural cells and tumor-vessel regression [55]. The blockade of both VEGF and PDGF receptor-*β* signaling in tumor blood vessels with kinase inhibitors also induces tumor vessel regression [56, 57], and paracrine signaling between pericytes and the tumor endothelium [58] could increase the instability of the tumor vessels of FoxC2+/– mice by reducing VEGF and PDGF-B signaling [15].

## 5. FoxC2 in Cancer Cells

Tumor FoxC2 expression is not restricted to the vasculature (Table 1) [15]. T-MTA-6A tissue-array (NCI/NIH) analysis revealed the localization of FoxC2 protein in the majority of breast adenocarcinomas, including lobular and ductal adenocarcinoma, and in about half of colonic adenocarcinomas [15]. FoxC2 expression has also been reported in esophageal cancer cells [39], and the survival rate is significantly lower for patients with high levels of FoxC2 expression than for patients with low levels of FoxC2 expression, which suggests that FoxC2 could be used as a novel, independent prognosis factor for patients with esophageal cancer [39] (Table 1). Patients with high levels of FoxC2 expression also have higher levels of MMP2 and MMP9 expression [39].

FoxC2 is an important regulator of EMT, a key process that is often activated during tumor progression and metastasis [59]. FoxC2 expression is significantly correlated with highly aggressive basal-like breast cancers, and FoxC2 overexpression increases the metastatic potential of mouse mammary carcinoma cells to the lung [40]. The EMT-inducing transcription factors Twist, Snail, and Goosecoid, as well as TGF-*β*, which also induces EMT, can induce FoxC2 expression in epithelial cells [40, 60], and FoxC2 overexpression promotes mesenchymal differentiation, as well as the induction of MMP2 and MMP9 expression [40]. Furthermore, FoxC2 indirectly represses expression of the epithelial marker E-cadherin, whose loss is considered a hallmark of EMT [59], and directly downregulates p120-catenin, a regulatory protein that stabilizes E-cadherin at the adhesion junctions of epithelial cells [61]. Thus, accumulating evidence indicates that EMT-inductive signaling molecules, such as TGF-*β*, stimulate FoxC2 expression in carcinoma cells along with other EMT-promoting transcription factors and mediates mesenchymal differentiation, thereby leading to EMT and metastasis [59]. EMT has also been linked to the genesis of cancer stem cells [62]; for example, mammary epithelial cells have been shown to acquire properties associated with breast cancer stem cells, including the CD44^high^/CD24^low^ signature, after undergoing EMT and to express EMT-inducing transcription factors such as FoxC2 (Table 1) [41]. Notably, tumor cells undergoing EMT acquire a migratory phenotype by ectopically expressing mesenchymal genes, including FoxC2, which first function in the developing embryo, and tumor cells may adopt many other developmental signaling pathways that function in their ancestral precursor cells [63–65]. Thus, EMT may represent a critical point of convergence between the signaling paradigms that regulate development and tumor metastasis.

## 6. Future Perspectives

Though we are just beginning to understand how FoxC2 contributes to cancer development and related pathological conditions, its newly discovered role in tumor angiogenesis and metastasis suggests that FoxC2 could be an intriguing target for anticancer therapies. Drug therapies rarely target transcription factors directly, but a combination of advanced technologies, such as systems biology and computational chemistry, could lead to the development of viable transcriptional approaches for preventing cancer development and progression, as well as other conditions that involve pathological angiogenesis. For example, FoxM1 is currently being investigated as a target for cancer therapy [66], and several chemical inhibitors, including proteasome inhibitors, of FoxM1 have been reported [67–71], as well as a cell-penetrating peptide inhibitor of FoxM1 function [72]. The precise function of FoxC2 in the tumor microenvironment, including both cancer cells and endothelial cells, must be better understood before the development of FoxC2-based therapies can be considered and, consequently, studies focusing on the mechanisms controlled by FoxC2 are necessary. The identification of relatively small peptides that can selectively block the function of FoxC2 would also be a useful step toward designing FoxC2-specific inhibitors.

## Figures and Tables

**Table 1:** Summary of FoxC2 expression and prognosis in human cancer.

Tumor type	Number	FoxC2 expression	Prognosis
Melanoma [15]		Vascular endothelial cells	Not evaluated
Breast, colon [15]		Adenocarcinoma	Not evaluated
Esophageal [39]	70	Cytoplasm of cancer cells	5-year survival: 70% FoxC2 (low); 30% FoxC2 (high)
Breast [40]	18	Highly aggressive basal-like tumors	Not evaluated
Breast [41]		CD44^high^/CD24^low^cells	Not evaluated
